# Helmoltz problem for the Riccati equation from an analogous Friedmann equation

**DOI:** 10.1140/epjc/s10052-021-09966-0

**Published:** 2022-01-07

**Authors:** Valerio Faraoni

**Affiliations:** Department of Physics and Astronomy, Bishop’s University, 2600 College Street, Sherbrooke, QC J1M 1Z7 Canada

## Abstract

We report a solution of the inverse Lagrangian problem for the first order Riccati differential equation by means of an analogy with the Friedmann equation of a suitable Friedmann–Lemaître–Robertson–Walker universe in general relativity. This analogous universe has fine-tuned parameters and is unphysical, but it suggests a Lagrangian and a Hamiltonian for the Riccati equation and for the many physical systems described by it.

## Introduction

The inverse variational problem, or Helmoltz problem, for a set of ordinary differential equations consists of finding a Lagrangian such that the associated Euler–Lagrange equations reproduce the given system. Although necessary and sufficient conditions for solving the inverse variational problem were already given by Helmoltz in 1887 [1] they are rather involved [2, 3] and, in general, determining whether they are satisfied and finding the explicit solution is cumbersome and may require one to solve a large system of equations (e.g., [4, 5]).

An alternative, unconventional, approach to the Helmoltz problem is provided by analogies: if a certain differential equation is analogous to another one ruling an analogous system which admits a known Lagrangian or Hamiltonian formulation, the association of a Lagrangian with the original equation is straightforward. Of course, this approach can only be applied to systems analogous to other systems with known Lagrangian or Hamiltonian formulations, that is, in exceptional cases. Here we consider the first order non-linear Riccati equation [13, 14]

1 $$\begin{aligned} \frac{du(x)}{dx} + c_0 \,u^2(x) +c_1=0 \,, \end{aligned}$$

where $c_{0,1}$ are constants, which describes many physical systems including, e.g., falling raindrops [21, 22] or charges in constant electric field, avalanches [15], debris slides [16], geomagnetic fields [17], and box models of ocean basins [18]; the Riccati equation is also related to the Schrödinger, the Ermakov-Pinney, and other equations of fundamental physics [19, 20]. A second order equation is naturally associated with Eq. (1): its generalizations, including systems of non-linear oscillators, and their Lagrangian formulations have been studied, also in relation with integrability and superintegrability [6–9]. Here, in a different context, we solve the Helmoltz problem of Eq. (1) by means of an analogous Friedmann equation. The latter describes a suitable universe in spatially homogeneous and isotropic (or Friedmann–Lemaître–Robertson–Walker, in short FLRW) cosmology, for which a Lagrangian is known. In order for the analogy to hold, one must impose a fine-tuned relation between the parameters of the analogous cosmos (equation of state parameter, energy density of the cosmic fluid, cosmological constant, and curvature index), hence the analogous universe is not physically relevant *per se*. However, this is not an issue here since we are not attempting to describe the real universe, but we are interested in solving the inverse Lagrangian problem for the Riccati equation (1).

The next section discusses in detail the analogy between Riccati and Friedmann equations; Sect. 3 exploits this analogy to solve the Helmoltz problem for the Riccati equation, while Sect. 4 contains some concluding remarks. We adopt the notation of Ref. [10]: the metric signature is ${-}{+}{+}{+}$, *G* is Newton’s constant, and units are used in which the speed of light is unity.

## A cosmological analogy for the Riccati equation

Spatially homogeneous and isotropic cosmologies are described by the FLRW line element

2 $$\begin{aligned} ds^2 =-dt^2 + a^2(t) \left( \frac{dr^2}{1-Kr^2} +r^2 d\Omega _{(2)}^2 \right) \end{aligned}$$

in comoving polar coordinates $\left( t,r,\vartheta , \varphi \right)$, where $d\Omega _{(2)}^2 = d\vartheta ^2 + \sin ^2 \vartheta \, d\varphi ^2$ is the line element on the unit 2-sphere, *K* is the curvature index normalized to $0, \pm 1$, and *a*(*t*) is the scale factor describing the expansion history of the universe [10, 11]. We assume that the latter is filled with a perfect fluid with energy density $\rho (t)$ and isotropic pressure *P*(*t*) related by the barotropic, linear, and constant equation of state

3 $$\begin{aligned} P=w\rho \,, \quad \quad w=\text{ const. } \end{aligned}$$

The evolution of the scale factor *a*(*t*) and of $\rho (t)$ and *P*(*t*) is ruled by the Einstein-Friedmann equations [10, 11]

4 $$\begin{aligned} \left( \frac{ \dot{a} }{a} \right) ^2 = \frac{8 \pi G}{3} \, \rho -\frac{K}{a^2} +\frac{\Lambda }{3} \,, \end{aligned}$$

5 $$\begin{aligned} \frac{\ddot{a}}{a} =-\frac{4\pi G}{3} \left( \rho +3P\right) +\frac{\Lambda }{3} \,, \end{aligned}$$

6 $$\begin{aligned} \dot{\rho } +3H \left( P+\rho \right) =0 \,, \end{aligned}$$

where an overdot denotes differentiation with respect to the cosmic (or “comoving”) time *t* and $\Lambda$ is the cosmological constant.

It is well-known [12, 30, 31] that by combining the Friedmann equation (4) and the acceleration Eq. (5) written in terms of the conformal time $\eta$ (defined by $dt\equiv a d\eta$), one obtains a Riccati equation (1) (a similar coordinate transformation has been known for the two-body problem since the times of Euler [32–37]). Here we pose instead the question of whether the Friedmann equation *in cosmic time* can assume the Riccati form (1). The answer is affirmative, but this only happens when the fluid has (phantom) equation of state parameter $w=-5/3$, hyperbolic spatial sections with $K=-1$, and (fine-tuned) cosmological constant $\Lambda \ne 0$, or when $w=1/3$, $K=-1$, and $\Lambda >0$. We derive this result in the following.

Assuming the equation of state (3), the covariant conservation Eq. (6) is integrated to [10, 11]

7 $$\begin{aligned} \rho (a) = \frac{\rho _0}{ a^{3(w+1)}} \,, \end{aligned}$$

where $\rho _0>0$ is a constant. Then the Friedmann equation (4) is recast as

8 $$\begin{aligned} \dot{a} = \pm \sqrt{ \frac{8\pi G}{3} \, \rho _0 a^{-(3w+1)} - K +\frac{\Lambda }{3} \, a^2} \,, \end{aligned}$$

where the argument of the square root is necessarily non-negative if the Friedmann equation (4) is to admit solutions. We now ask when this argument is a perfect square: there are three possibilities for this to happen. The first case corresponds to $K=-1$ and $w=-5/3$ and allows one to write the argument of the square root as

9 $$\begin{aligned} \left( \sqrt{ \frac{8\pi G \rho _0}{3} } a^2 \right) ^2 + (\sqrt{1})^2 +\frac{\Lambda }{3}\, a^2 \,; \end{aligned}$$

then we set $\frac{ \Lambda a^2}{3} = \pm 2 \sqrt{1} \sqrt{\frac{8\pi G \rho _0}{3} } \, a^2$, which is satisfied only by tuning the cosmological constant to one of the two values

10 $$\begin{aligned} \Lambda =\pm 6 \sqrt{\frac{8\pi G \rho _0}{3}} \end{aligned}$$

and then

11 $$\begin{aligned} \dot{a} = \pm \left( \sqrt{ \frac{8\pi G \rho _0}{3}} \, a^2 \pm 1 \right) \,, \end{aligned}$$

where the two ± signs are independent, *i.e.*, there are four possible solutions here.

The second possibility appears for $K=-1$, $w=1/3$, and $\Lambda >0$. In this case we identify

12 $$\begin{aligned} \frac{8\pi G \rho _0}{3 a^2} + 1 +\frac{\Lambda }{3} \, a^2&= \left( \sqrt{ \frac{8\pi G \rho _0}{3} } \, \frac{1}{a} \right) ^2 + \left( \sqrt{ \frac{\Lambda }{3} } \, a \right) ^2 \nonumber \\&\quad + 2 \sqrt{ \frac{8\pi G\rho _0 \Lambda }{9}} \nonumber \\&= \left( \sqrt{ \frac{8\pi G \rho _0}{3} } \, \frac{1}{a} +\sqrt{\frac{\Lambda }{3} } \, a \right) ^2 \end{aligned}$$

provided that

13 $$\begin{aligned} \rho _0 \Lambda = \frac{9}{32\pi G} \,, \end{aligned}$$

and then

14 $$\begin{aligned} \dot{a} = \pm \left( \sqrt{ \frac{8\pi G \rho _0}{3}} \, \frac{1}{a} +\frac{1}{2} \sqrt{ \frac{3}{8\pi G \rho _0} } \, a \right) \,. \end{aligned}$$

The third possibility occurs for $K=+1$, $w=1/3$, and $\Lambda =\frac{9}{32\pi G \rho _0} >0$, which yields the identification

15 $$\begin{aligned} \frac{8\pi G \rho _0}{3 a^2} - 1 +\frac{\Lambda }{3} \, a^2&= \left( \sqrt{ \frac{8\pi G \rho _0}{3} } \, \frac{1}{a} \right) ^2 + \left( \sqrt{ \frac{\Lambda }{3} } \, a \right) ^2 \nonumber \\&\quad - 2 \sqrt{ \frac{8\pi G\rho _0 \Lambda }{9}} \nonumber \\&= \left( \sqrt{ \frac{8\pi G \rho _0}{3} } \, \frac{1}{a} -\sqrt{\frac{\Lambda }{3} } \, a \right) ^2 \,, \end{aligned}$$

therefore,

16 $$\begin{aligned} \dot{a} = \pm \left( \sqrt{ \frac{8\pi G \rho _0}{3} } \, \frac{1}{a} - \frac{1}{2} \, \sqrt{ \frac{3}{8\pi G \rho _0} } \, a \right) \,. \end{aligned}$$

These fine-tunings between $\Lambda$ and the initial condition on the energy density ($\rho _0$) are clearly unphysical and the discussion is purely of mathematical interest unless some physical mechanism is found that achieves the tuning, which seems unlikely.

The differential equations for the scale factor can be solved directly by quadratures, but here we reduce them to Riccati equations because our goal is to solve the Helmoltz problem for the Riccati equation.

### $\Lambda = \pm 4 \sqrt{6\pi G\rho _0}$

In the first case, we have the equation

17 $$\begin{aligned} \dot{a} = \pm \left( \sqrt{ \frac{8\pi G}{3} \, \rho _0 } \, a^2 \pm 1 \right) \,, \end{aligned}$$

where the two ± signs are independent, *i.e.*, there are four equations. Consider first the two possibilities resulting from the equation

18 $$\begin{aligned} \dot{a} = \pm \sqrt{ \frac{8\pi G}{3} \, \rho _0 } \, a^2 + 1 \,, \end{aligned}$$

which is of the Riccati form (1) with

19 $$\begin{aligned} c_0= & {} \mp \sqrt{\frac{8\pi G}{3} \, \rho _0} \,,\end{aligned}$$

20 $$\begin{aligned} c_1= & {} -1 \,. \end{aligned}$$

This Riccati equation is solved by setting [13, 14]

21 $$\begin{aligned} a(t) \equiv \frac{1}{c_0} \, \frac{ \dot{v}}{v} \,, \end{aligned}$$

which yields

22 $$\begin{aligned} \ddot{v} + c_0 \, c_1 v = 0 \,. \end{aligned}$$

We discuss separately the two possibilities corresponding to upper and lower sign for $c_0$.

#### Upper sign

By choosing the upper sign we have a positive (and fine-tuned) cosmological constant $\Lambda$, $c_0 \, c_1>0$, and the solution of the resulting harmonic oscillator Eq. (22) is $v(t)= A \sin \left( \sqrt{c_0 \, c_1} \, t \right) + B \cos \left( \sqrt{c_0 \, c_1} \, t \right)$ where *A* and *B* are integration constants, yielding

23 $$\begin{aligned} a(t) =\sqrt{ \frac{c_1}{c_0} } \, \frac{ B \sin \left( \sqrt{c_0 \, c_1} \, t \right) -A \cos \left( \sqrt{c_0 \, c_1} \, t \right) }{ A\sin \left( \sqrt{c_0 \, c_1} \, t \right) +B \cos \left( \sqrt{c_0 \, c_1} \, t \right) } \,, \end{aligned}$$

where *a*(*t*) must be non-negative. Although there are two arbitrary integration constants *A* and *B* for Eq. (22), in practice there is only one arbitrary initial condition *A*/*B* or *B*/*A*, corresponding to the fact that the equivalent Riccati equation is of first order. Special initial conditions give particular solutions.

- $A=0$, $B\ne 0$ In this case the solution (23) becomes
  24 $$\begin{aligned} a(t) = \left( \frac{3}{8\pi G \rho _0} \right) ^{1/4} \tan \left[ \left( \frac{8\pi G \rho _0}{3} \right) ^{1/4} \, t \right] \end{aligned}$$
  in the range
  25 $$\begin{aligned} 0\le t <\frac{\pi }{2} \left( \frac{3}{8\pi G \rho _0} \right) ^{1/4} \equiv t_* \,, \end{aligned}$$
  which represents a universe starting at a Big Bang and ending in a Big Rip ($a\rightarrow +\infty$) at a finite future $t_*$. Here we denote as “Big Bang” a zero of the scale factor $a(t) \rightarrow 0$, but this word does not have the usual textbook meaning in the sense that the derivative $\dot{a}$, the energy density $\rho$, and the pressure *P* do not diverge at this “Big Bang”. Here the singularity is “soft” in the sense that $\dot{a}(0)$ is finite (but the Hubble function $H(t)\equiv \dot{a}/a$ still diverges). Likewise, the Big Rip singularity is not an inverse power-law, as it would happen if only the phantom fluid were present [28, 29], but has an unusual tangent-like divergence.
- $A\ne 0$, $B=0$ The solution (23) becomes
  26 $$\begin{aligned} a(t) = - \left( \frac{3}{8\pi G \rho _0} \right) ^{1/4} \cot \left[ \left( \frac{8\pi G \rho _0}{3} \right) ^{1/4} \, t \right] \end{aligned}$$
  in the range
  27 $$\begin{aligned} t_*<t < 2 t_* \,; \end{aligned}$$
  there are again a Big Bang (in the sense that $a=0$ at $t=t_*$) and a Big Rip where $a\rightarrow +\infty$ as $t\rightarrow 2t_*$.
- $A=B \ne 0$ In this case the solution (23) takes the form
  28 $$\begin{aligned} a(t) = - \left( \frac{3}{8\pi G \rho _0} \right) ^{1/4} \frac{ \cos \left[ 2\left( \frac{8\pi G \rho _0}{3} \right) ^{1/4} \, t \right] }{ 1+\sin \left[ 2\left( \frac{8\pi G \rho _0}{3} \right) ^{1/4} \, t \right] } \end{aligned}$$
  in the range
  29 $$\begin{aligned} \frac{t_*}{2}<t < \frac{3 t_*}{2} \,. \end{aligned}$$
  Again, the solution begins in a “soft” Big Bang (in the sense that $a(t)\rightarrow 0$) and ends in a Big Rip singularity $a\rightarrow +\infty$.

#### Lower sign

In this case $\Lambda <0$, $c_0>0$, $c_1<0$, and $v(t) = A\text{ e}^{ \sqrt{c_0|c_1|} \, t} +B \text{ e}^{ -\sqrt{c_0|c_1|} \, t}$ (with *A*, *B* integration constants), yielding

30 $$\begin{aligned} a(t) = \sqrt{ \frac{|c_1|}{c_0} } \, \frac{ A\text{ e}^{ \sqrt{c_0|c_1|} \, t} -B \text{ e}^{ -\sqrt{c_0|c_1|} \, t} }{ A\text{ e}^{ \sqrt{c_0|c_1|} \, t} +B \text{ e}^{ -\sqrt{c_0|c_1|} \, t} }\,. \end{aligned}$$

Contrary to the previous situation (upper sign in Eq. (22)), for $A= 0$, $B\ne 0$ the scale factor is negative, therefore we discard this possibility and we assume that $A\ne 0$. Special initial conditions include the following.

- $A\ne 0$, $B=0$ In this case the solution is the static universe with
  31 $$\begin{aligned} a(t) = \sqrt{ \frac{|c_1|}{c_0} } = \left( \frac{3}{8\pi G \rho _0} \right) ^{1/4} \equiv a_* \end{aligned}$$
  resulting from the balance of the negative cosmological constant with the repulsive phantom fluid and the curvature term in the Friedmann equation (4). The general solution (30) with $A\ne 0$ asymptotes to $a_*$ at late times $t\rightarrow +\infty$, irrespective of the value of this integration constant, therefore the solution $a(t) \equiv a_*$ is stable with respect to homogeneous perturbations and is a late-time attractor in the phase space of the solutions.
- $A=B \ne 0$ With this choice of initial conditions, the scale factor is
  32 $$\begin{aligned} a(t)= & {} \sqrt{ \frac{|c_1|}{c_0} } \, \tanh \left( \sqrt{|c_1|c_0} \, t \right) \nonumber \\= & {} a_* \, \tanh \left[ \left( \frac{8\pi G \rho _0}{3} \right) ^{1/4} \, t \right] \,. \end{aligned}$$
  This universe begins from a Big Bang (again, in the sense $a\rightarrow 0$) at $t=0$ and evolves for an infinite time, with the scale factor asymptoting to $a_*$ as $t\rightarrow + \infty$. In this case *a*(*t*) is analogous to the speed of a raindrop falling vertically from rest in a constant gravitational field and reaching terminal speed [21, 22], as $a(t) \simeq a_*$ when $t\rightarrow +\infty$.
- $A=-B \ne 0$ In this case, the scale factor
  33 $$\begin{aligned} a(t) = \left( \frac{3}{8\pi G \rho _0} \right) ^{1/4} \coth \left[ \left( \frac{8\pi G \rho _0}{3} \right) ^{1/4} \, t \right] \end{aligned}$$

corresponds to the unusual contracting branch of a pole of the scale factor *a*(*t*), where the universe begins from infinite size at $t=0$ and decreases monotonically, asymptoting to the constant value $a_*$ as $t\rightarrow +\infty$. Again, the static universe (31) is a late-time attractor in phase space.

#### The remaining sign possibilities

In this case we have

34 $$\begin{aligned} \dot{a} = \pm \left( \sqrt{ \frac{8\pi G\rho _0}{3}} \, a^2 -1 \right) \,, \end{aligned}$$

which has the solution

35 $$\begin{aligned} a(t) = \pm \left( \frac{3}{8\pi G \rho _0} \right) ^{1/4} \arctan \left[ \left( \frac{8\pi G \rho _0}{3} \right) ^{1/4} \, (t-t_0)\right] \nonumber \\ \end{aligned}$$

where the range of *t* is chosen so that *a*(*t*) is non-negative.

### $\rho _0 \Lambda = 9/(32\pi G)$

We have two possibilities corresponding to this fine-tuned choice of $\rho _0$. In the first case, Eq. (8) assumes the form

36 $$\begin{aligned} \dot{a} = \pm \left( \frac{ \sqrt{A}}{a} + \frac{a}{2\sqrt{A}} \right) \,, \end{aligned}$$

where $A\equiv \sqrt{ 8\pi G \rho _0/3}$. This is is not a Riccati equation and is readily integrated, giving the solution

37 $$\begin{aligned} a_{( \pm )} (t) = \sqrt{ \text{ e}^{ \pm \, \frac{(t-t_0)}{\sqrt{A} }} -2A } \,, \end{aligned}$$

where $t_0$ is an integration constant.

In the second case, Eq. (8) becomes

38 $$\begin{aligned} \dot{a} = \pm \left( \frac{ \sqrt{A}}{a} - \frac{a}{2\sqrt{A}} \right) \,, \end{aligned}$$

which admits the solutions

39 $$\begin{aligned} a_{( \pm )} (t) = \sqrt{ 2A - \text{ e}^{ \mp \,\frac{(t-t_0)}{\sqrt{A}} } } \,. \end{aligned}$$

## Solving the inverse Lagrangian problem for the Riccati equation

We only need one of the cosmological solutions to find a Lagrangian for the Riccati equation (1). Based on the cosmological analogy illustrated in the previous section, focus on the case in which the Riccati equation (1) is the same as the Friedmann equation for a FLRW universe permeated by a perfect phantom fluid with $P=-5\rho /3$, $K= -1 = -c_1^2$, and $\Lambda = 6 \,c_0 \, c_1$. This cosmic analogy inspires an unconventional solution of the inverse Lagrangian (or Helmoltz) problem of finding a Lagrangian and a Hamiltonian for the Riccati equation (1). The standard Lagrangian for FLRW cosmology^1^ [23–27]

40 $$\begin{aligned} L \left( a, \dot{a} \right) = a\dot{a}^2 +\frac{8\pi G}{3} \, a^3 \rho -Ka + \frac{\Lambda }{3} \, a^2 \end{aligned}$$

suggests to use

41 $$\begin{aligned} L_1 \left( u, \dot{u} \right) = u\dot{u}^2 +c_0^2 u^5 +c_1^2 u + 2 \,c_0 \, c_1 u^2 \end{aligned}$$

as a Lagrangian for the Riccati equation. The corresponding Hamiltonian is

42 $$\begin{aligned} \mathcal{H}_1 = \dot{u} \, \frac{\partial L_1}{\partial \dot{u}} -L_1= u\dot{u}^2 -c_0^2 u^5 -c_1^2 u -2 \, c_0 \, c_1 u^2 \,. \end{aligned}$$

Since this Hamiltonian does not depend explicitly on time it is conserved, yielding the Beltrami identity $\mathcal{H}_1=$ const. To actually reproduce the Riccati equation, one must choose this constant to be zero, according to the fact that the dynamics of general relativity is constrained [10]. In cosmology, this fact is reflected in the fact that the Friedmann equation is a first order constraint, not a full (second order) equation of motion, and the vanishing of $\mathcal{H}_1$ enforces precisely this constraint (“Hamiltonian constraint”) [10, 11]. Setting $\mathcal{H}_1=0$ yields

43 $$\begin{aligned} \dot{u}= \pm \left( c_0 \, u^2 +c_1 \right) \,. \end{aligned}$$

Choosing the lower sign reproduces the Riccati equation (1), while choosing the upper sign reproduces the same equation with the exchange $\left( c_0, c_1 \right) \rightarrow \left( -c_0, - c_1 \right)$. Since $c_{0,1}$ are arbitrary non-zero coefficients, this sign change is immaterial.

In actual fact, one can use the simpler Lagrangian

44 $$\begin{aligned} L_2 \left( u, \dot{u} \right) = \dot{u}^2 +c_0^2 u^4 +c_1^2 + 2 \, c_0 \, c_1 u \end{aligned}$$

and the associated Hamiltonian

45 $$\begin{aligned} \mathcal{H}_2 = \dot{u}^2 -c_0^2 u^4 -c_1^2 - 2 \, c_0 \, c_1 u \,. \end{aligned}$$

Again, setting $\mathcal{H}_2=0$ reproduces the Riccati equation (1).

The equation $\mathcal{H}_2=0$ lends itself to a new analogy with point particle mechanics;^2^ it can be seen as the energy conservation equation for a particle of unit mass in one-dimensional motion along the *u*-axis in the potential energy

46 $$\begin{aligned} V(u) = - c_0 \, u \left( \frac{c_0}{2} \, u^3 + c_1 \right) \end{aligned}$$

and with kinetic energy $\dot{u}^2/2$. This energy conservation equation is

47 $$\begin{aligned} \frac{\dot{u}^2}{2} +V(u) = E =\frac{ c_1^2}{2} \end{aligned}$$

for the special value of the total mechanical energy $E=c_1^2/2 > 0$.

Consider first the case $c_0 \, c_1>0$: then the potential *V*(*u*) has an absolute maximum

48 $$\begin{aligned} V_\mathrm {max}= \frac{ 3 \, c_0^{2/3} c_1^{4/3}}{2^{7/3}} > 0 \end{aligned}$$

at $u_\mathrm {max}= \mp \left| \frac{c_1}{2\, c_0}\right| ^{1/3} <0$ (the sign of $u_\mathrm {max}$ depends on the signs of $c_0$ and $c_1$). The motion is always unbounded: there are no turning points if $V_\mathrm {max} \le E$, corresponding to $\frac{c_0}{c_1} \le \frac{4}{\sqrt{27}}$, and there are two turning points otherwise. The same conclusion is reached for $c_0 \, c_1 <0$.

## Concluding remarks

The Riccati equation describes several physical systems, for example the vertical speed *v*(*t*) of a falling raindrop subject to gravity and friction quadratic in the velocity (e.g., [21, 22]). Let *g* be the constant acceleration of gravity and consider a vertical axis pointing downwards, then Newton’s second law is

49 $$\begin{aligned} m \, \frac{dv}{dt} = mg-\alpha \, v^2 \,, \end{aligned}$$

where *m* is the mass of the drop and $\alpha$ is a friction coefficient. The function *v*(*t*) satisfies the Riccati equation $\dot{v} +\frac{\alpha }{m} \, v^2 -g=0$, and one would naively think that it is sufficient to write down the Lagrangian for this particle to obtain the Lagrangian for the Riccati equation, but including quadratic (or, in general, non-linear) friction in the Lagrangian formalism is not so easy [38]. Indeed, the Riccati Lagrangians $L_1$ or $L_2$ provided by Eqs. (41) and (44) solve this problem of physical interest, as well as that of the many physical systems described by Eq. (1), even though the analogous universe is essentially of no relevance for physical cosmology. The Friedmann equation describing this universe is formally a Riccati equation, but this cosmos is unphysical because its parameters must be tuned in order for the analogy to hold (the cases studied are the only ones for which the Friedmann equation *in cosmic time* assumes the Riccati form (1)). This is not an issue here since our goal is not to describe the real universe with a Riccati equation (which is usually done by rewriting a combination of the Friedmann equation and of the acceleration equation in conformal time [12, 30, 31]), but rather to solve the Helmoltz problem for Eq. (1). The explicit Lagrangian and Hamiltonian for the Riccati equation are very simple, but one could not guess them without the analogy.

## Data Availability

This manuscript has no associated data or the data will not be deposited. [Authors’ comment: There are no data associated with this work because of its entirely theoretical nature.]

## Footnotes

1 The Lagrangian can also be obtained by using the lapse function (one obtains the Friedmann equation by varying the action with respect to the lapse function).

2 The Hamiltonian $\mathcal{H}_1$ lends itself to an analogy with the one-dimensional motion of a particle with mass dependent on the position, which is not as compelling, and this is the reason why we switch to the Lagrangian $L_2$ instead of using $L_1$ in this mechanical analogy.
